# MK2 drives progression of pancreas and colon cancers by suppressing CD8^+^ T cell cytotoxic function and is a potential immunotherapy target

**DOI:** 10.3389/fimmu.2023.1212100

**Published:** 2023-06-21

**Authors:** Damian Jacenik, Eric J. Lebish, Ellen J. Beswick

**Affiliations:** ^1^ Department of Cytobiochemistry, Faculty of Biology and Environmental Protection, University of Lodz, Lodz, Poland; ^2^ Division of Gastroenterology, Department of Internal Medicine, University of Utah, Salt Lake City, UT, United States; ^3^ Division of Digestive Diseases and Nutrition, Department of Internal Medicine, University of Kentucky, Lexington, KY, United States

**Keywords:** mitogen-activated protein kinase-activated protein kinase 2, MAPKAPK2, MK2, cytotoxic T cell, CD8^+^ T cell, pancreas cancer, colon cancer, tumorigenesis

## Abstract

**Background:**

Immune cell composition is a critical and dynamic component of the tumor microenvironment, which has an impact on immunosuppression and progression of cancer. T cells, especially CD8^+^ T cells, are one of the major immune cell types responsible for tumor cell killing employing receptor-ligand mediated apoptosis and/or releasing lytic granules among others. Accumulating evidence highlighted that adoptive transfer of activated and/or modified immune cells can enhance anti-tumorigenic immune responses and serve as promising therapy approach for patients with cancers. The mitogen-activated protein kinase-activated protein kinase 2 (MK2) is a serine/threonine protein kinase, which controls production and secretion of numerous pro-inflammatory cytokines and chemokines involved in tumorigenesis. However, limited efforts have been made to learn how MK2 may affects CD8^+^ T cell action and function in the tumor microenvironment especially in gastrointestinal cancers.

**Methods:**

To explore the therapeutic potential of MK2 in the immune response mediated by CD8^+^ T cells, RAG1 knockout mice with PK5L1940 and BRAF cells-derived allograft tumors were treated with WT or MK2 knockout CD8^+^ T cells. The phenotype of CD8^+^ T cells with MK2 depletion were evaluated *in vitro*. Immunofluorescence staining, real-time PCR and multiplex analysis were utilized to estimate the expression of apoptotic and lytic factors.

**Results:**

Here, we show that CD8^+^ T cells with MK2 depletion prevent gastrointestinal cancer growth, which is accompanied by enhanced expression and secretion of factors related to apoptosis. Moreover, using *in vitro* and *in vivo* approaches, we found that depletion of MK2 lead to hyperactivation of CD8^+^ T cells and enhanced anti-tumor immunity.

**Conclusion:**

Overall, we documented that MK2 drives the progression of gastrointestinal cancers and prevents immune response generated by CD8^+^ T cells suggesting potential implications of MK2 in the immunotherapy of gastrointestinal cancers.

## 1 Introduction

The mitogen-activated protein kinase-activated protein kinase 2 (MK2) is a serine/threonine protein kinase, which is regulated by p38 MAPK, but also functions independently of p38 signaling (1). Studies indicated the significance of p38 in immune response where it is involved in the production of multiple mediators of inflammation, the most well-known of which are interleukin (IL) -1, tumor necrosis factor-α (TNF-α) and cyclooxygenase-2 (2, 3).

MK2 plays a multifunctional role in pathophysiology of diseases like pulmonary fibrosis, cardiovascular diseases, and cancer progression such as head and neck cancers, colon, breast or bladder cancer (4–9). Nevertheless, modulation of the immune response by MK2 has growing attention and the ability of MK2 to regulate cytokine and chemokine production may be critical in the context of immunotherapy for patients with cancers. In fact, *in vitro* and *in vivo* experiments show that the levels of cytokines such as IL-1β, IL-6, IL-10, interferon-γ (IFN- γ) and TNF-α, and chemokines such as monocyte chemoattractant protein-1 (MCP-1), macrophage inflammatory protein (MIP) -1α and MIP- 2α are dependent on MK2 activity (10–14). Moreover, according to pre-clinical studies, MK2 inhibition leads to reduced proliferation, migration and invasion of cancer cells. Furthermore, several processes including angiogenesis, DNA synthesis, cell cycle of cancer cells, and DNA damage response machinery are regulated by MK2 signaling (4, 5, 7, 15, 16).

However, the effect of MK2 expression and signaling on immune cell activity is poorly explored. There is evidence indicating the impact of MK2 on macrophage activity, function and phenotype, which impacts cancer progression. Studies have shown that MK2 is able to promote polarization of the above-mentioned myeloid cells into the pro-tumorigenic M2 phenotype of macrophages (17). In fact, our group highlighted how MK2 functions in the immune response mediated by macrophages in the development of pancreatic neuroendocrine tumors (13). On the other hand, Soukup et al. found that mice with MK2 knockout (KO) are characterized by suppressive phenotype of dendritic cells in melanoma (18). Interestingly, it was noted that depletion of p38 in T cells enhances regulatory T cell induction suggesting that not only p38, but also MK2 may be involved in the modulation of T cell function (19).

We found that MK2 is critical for the activity of CD8^+^ T cell, and its action may promote progression of gastrointestinal cancers. To explore the therapeutic significance of MK2 on the pancreatic and colon tumorigenesis, and the impact of MK2 expression and signaling on gastrointestinal tumor microenvironment, we employed WT mice and animals with MK2 KO as well as gastrointestinal cancer cell-derived allograft tumor models. Additionally, *in vitro* analyses using WT and MK2 KO CD8^+^ T cells were conducted to explore the role of MK2 on CD8^+^ T cell function. Our novel findings suggest that depletion of MK2 in CD8^+^ T cells enhances cytotoxic activity and decreases tumor growth.

## 2 Materials and methods

### 2.1 Mice

MK2^tm1Mgl^ (MK2 KO) mice were obtained from Dr. Mathias Gaestel (Hannover Medical School, Germany). C57BL/6, i.e. wild type (WT) mice and B6.129S7-Rag1^tm1Mom/J^ (RAG1 KO) mice were obtained from Jackson Laboratory (Bar Harbor, MA, USA) and bred in-house. MK2 KO mice have been genotyped to confirm MK2 knockdown. The animals were housed in the Comparative Medicine Center, University of Utah Health, UT, USA at constant temperature (22 – 24°C), relative humidity ~55% and maintained under 12 h light/dark cycle (lights turned on 8 a.m.) with access to standard chow pellets and tap water *ad libitum*. The research has been approved by the University of Utah Institutional Animal Care and Use Committee.

### 2.2 Isolation and culture of CD8^+^ T cells

The CD8^+^ T cells were isolated from spleens obtained from 6 – 8-week-old WT and MK2 KO mice using Dynabeads™ Untouched™ Mouse CD8 Cells Kit (ThermoFisher Scientific, Waltham, MA, USA) according to manufacturer’s protocol where purity ranged from 92% – 95%. WT and MK2 KO CD8^+^ T cells were used for *in vitro* and *in vivo* experiments. Isolated WT and MK2 KO CD8^+^ T cells were plated and incubated in RPMI media (Corning, Tewksbury, MA, USA) supplemented with 10% heat-inactivated fetal bovine serum (ThermoFisher Scientific, Waltham, MA, USA), 1% penicillin/streptomycin (Corning, Tewksbury, MA, USA) and 1% L-glutamine (ThermoFisher Scientific, Waltham, MA, USA). The CD8^+^ T cells were activated with eBioscience™ cell stimulation cocktail (ThermoFisher Scientific, Waltham, MA, USA) for up to 24 h and collected for supernatant and real-time PCR analysis.

### 2.3 Murine allograft tumor models and adoptive transfer of CD8^+^ T cells

PK5L1940 cells provided by Dr. Michael Gough (Earle A. Chiles Research Institute, Portland, OR, USA) (20) and BRAF (BRAF^V600EΔTRZI^) cells provided by Dr. Daniel Worthley (South Australian Medical and Health Institute, Australia) (21) were grown in complete RPMI and DMEM media respectively; and used to generate murine allograft pancreas and colon tumor models. 1 × 10^6^ of PK5L1940 or 2 × 10^6^ of BRAF cells resuspend in PBS and mixed with Matrigel^®^ (Corning, Tewksbury, MA, USA) were injected into the flank of 6 – 8-week-old female RAG1 KO mice. Tumors were manually measured using a caliper starting from day 1. Some mice with PK5L1940 and BRAF cells derived allografts were administered peritumorally with 1 × 10^6^ of WT or MK2 KO CD8^+^ T cells resuspended in PBS. Tumor size was calculated according to the following formula: tumor size = $\frac{\left(\text{length }\times \text{ length }\times \text{ width}\right)}{2}$ .

### 2.4 RNA extraction, reverse transcription and real-time PCR

Tumor pieces and CD8^+^ T cells were homogenized in TRIzol™ reagent (ThermoFisher Scientific, Waltham, MA, USA) and RNA extraction was performed according to the manufacturer’s protocol. The quality and quantity of RNA were measured with a NanoDrop™ Lite Spectrophotometer (ThermoFisher Scientific, Waltham, MA, USA) and total RNA (100 ng/µL) was reversed transcribed using High-Capacity cDNA Reverse Transcription Kit (ThermoFisher Scientific, Waltham, MA, USA) with the following PCR settings: 25°C for 10 minutes, 37°C for 120 minutes and 85°C for 5 minutes. Quantitation of mRNA was performed using real-time PCR with validated FAM dye-labeled TaqMan^®^ probes (Applied Biosystems, Foster City, CA, USA) for *Actb* – Mm02619580_g1, *Casp3* – Mm01195085_m1, *Fas* – Mm01204974_m1, *Fasl* – Mm00438864_m1, *Gzmb* – Mm00442837_m1, *Lamp1* – Mm01217070_m1, *Prf1* – Mm00812512_m1. The reaction mixture consisted of cDNA, TaqMan^®^ Fast Advanced Master Mix (Applied Biosystems, Foster City, CA, USA), TaqMan^®^ Assays, and RNase-free water in a total volume of 10 μL. Cycle parameters for TaqMan^®^ assays were as follows: initial denaturation at 95°C for 3 min, followed by 40 cycles of sequential incubations at 95°C for 15 s and 60°C for 1 min. Results were normalized to the expression of housekeeping gene, i.e. *Actb*. All experiments were performed at least as duplicates on QuantStudio™ 5 Real-Time PCR System (ThermoFisher Scientific, Waltham, MA, USA). The endpoint used in real-time PCR quantification – CT – was defined as the PCR cycle number that crossed the signal threshold. Quantification of gene expression was performed using the comparative CT method (Sequence Detector User Bulletin 2; Applied Biosystems) and reported as the fold change relative to the mRNA of the mouse housekeeping gene.

### 2.5 Apoptosis assay

Tumors were divided into 4 mg pieces and homogenized using a bead beater. Homogenates were examined using the Apo-ONE^®^ Homogeneous Caspase-3/7 Assay (Promega, Madison, WI, USA) according to manufacturer’s instructions. Samples were examined for fluorescence using a SpectraMax Mini Plate Reader and Softmax Pro software.

### 2.6 Immunofluorescence analysis

Tumors were fixed in 4% paraformaldehyde, incubated in 15% and then 30% sucrose-PBS solutions for up to 12 h, each. Tumor pieces were embedded in the Tissue-Plus™ O.C.T. Compound Tissue-Plus™ (ThermoFisher Scientific, Waltham, MA, USA). Tumor sections (5μm) were blocked with 2% normal serum and incubated for 2 hours at room temperature with commercially available antibodies against CD8-Alexa Fluor™ 488 (clone 53-6.7, 1:200 dilution) and GZMB-PE (clone NGZB, 1:200 dilution) from ThermoFisher Scientific (Waltham, MA, USA) and BD Bioscience (San Diego, CA, USA), respectively. Subsequently, the sections were washed with PBS and mounted in SlowFade™ Gold Antifade Mountant with DAPI (ThermoFisher Scientific, Waltham, MA, USA). The tumor sections were imaged using EVOS™ M7000 Imaging System (ThermoFisher Scientific, Waltham, MA, USA) and double positive cells per field were calculated by the system software.

### 2.7 Multiplex analysis

PK5L1940 and BRAF cells-derived allograft tumors were divided into 8 mg pieces (± 0.5 mg) and incubated in RPMI media (Corning, Tewksbury, MA, USA) supplemented with 10% heat-inactivated fetal bovine serum (ThermoFisher Scientific, Waltham, MA, USA), 1% penicillin/streptomycin (Corning, Tewksbury, MA, USA) and 1% L-glutamine (ThermoFisher Scientific, Waltham, MA, USA) up to 18 h. Tumor culture supernatants were analyzed for CD137, GZMB and FAS in one custom array and IL-2, IL-10, and IFN-γ in another custom array (MilliporeSigma, Burlington, MA, USA) and Luminex^®^ in accordance with the manufacturer’s protocol.

### 2.8 Statistical analysis

Statistical analysis was performed using GraphPad Prism 8.0 (GraphPad Software Inc., San Diego, CA, USA). Results are presented as individual values with means ± standard deviation (SD). Unpaired t test, two-way ANOVA followed by Bonferroni’s multiple comparison *post hoc* test and ordinary one-way ANOVA followed by Tukey’s multiple comparison *post hoc* test were used for comparison of studied groups. *P* values < 0.05 was considered statistically significant.

## 3 Results

### 3.1 The progression of gastrointestinal cancers is suppressed by CD8^+^ T cells with MK2 depletion

**Figure 1:**
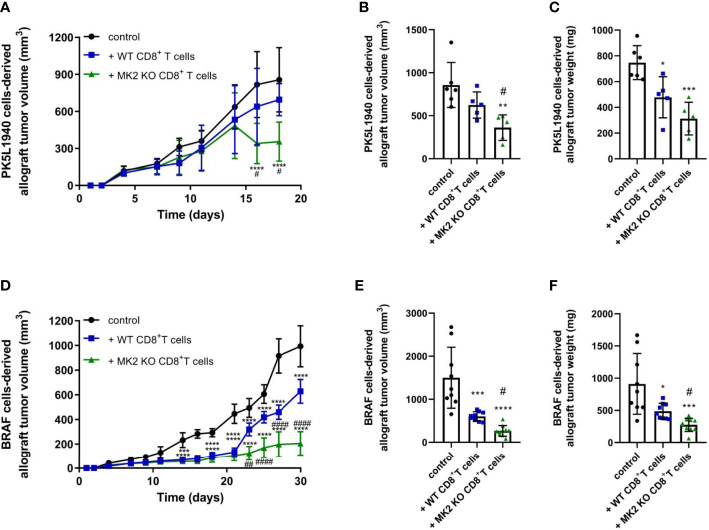
Tumors exposed to MK2 KO CD8^+^ T cells show decreased tumor growth in PK5L1940 and BRAF tumors shown by growth profile of tumors **(A, D)** and tumor volume **(B, E)** as well as tumor weight **(C, F)**. Data are presented as means ± SD; 5 – 10 mice per group; ^*^ *P* <  0.05, ^**^ *P* <  0.01, ^***^ *P* <  0.001, ^****^ *P* < 0.0001 vs. control; ^#^ *P*<  0.05, ^##^ *P* <  0.01, ^####^ *P* < 0.0001 vs. + WT CD8^+^ T cells.

Cytotoxic T cells are critical component of the immune response against cancer progression. Here, we set out to investigate the relevance of MK2 expression and signaling on CD8^+^ T cell function in the microenvironment of gastrointestinal cancers. To explore this goal, we employed PK5L1940 and BRAF cancer cells to compare results in both KRAS and BRAF mutant models in RAG1 KO mice. We identified that mice treated with MK2 KO CD8^+^ T cells are characterized by suppressed growth of pancreas tumors when compared to both control mice and mice treated with WT CD8^+^ T cells (**Figure 1A**). As was shown in **Figures 1B, C**, we observed that MK2 KO CD8^+^ T cells are able to reduce pancreas tumor volume and weight by approximately 50%. To confirm the effect of MK2 KO on cytotoxic potential of CD8^+^ T cells in the tumor microenvironment, colon cancer model was used as well. In mice with BRAF cells-derived allograft tumors, administration of WT and MK2 KO CD8^+^ T cells was related to decreased growth of tumors (**Figures 1D–F**). Nevertheless, should be noted that mice treated with MK2 KO CD8^+^ T cells are manifested by suppressed tumor growth compared not only to control mice, but also mice treated with WT CD8^+^ T cells (**Figures 1D–F**). The final evaluation of tumor volume and weight and further analysis of tumors were performed after 18 or 30 days of PK5L1940 or BRAF tumor growth, respectively based on tumor size. Overall, our data show that MK2 expression/signaling is key for CD8^+^ T cells and its action may affects the progression of gastrointestinal cancers.

### 3.2 MK2 depletion induces increased cytotoxic activity of CD8^+^ T cells and apoptosis in gastrointestinal tumors

**Figure 2:**
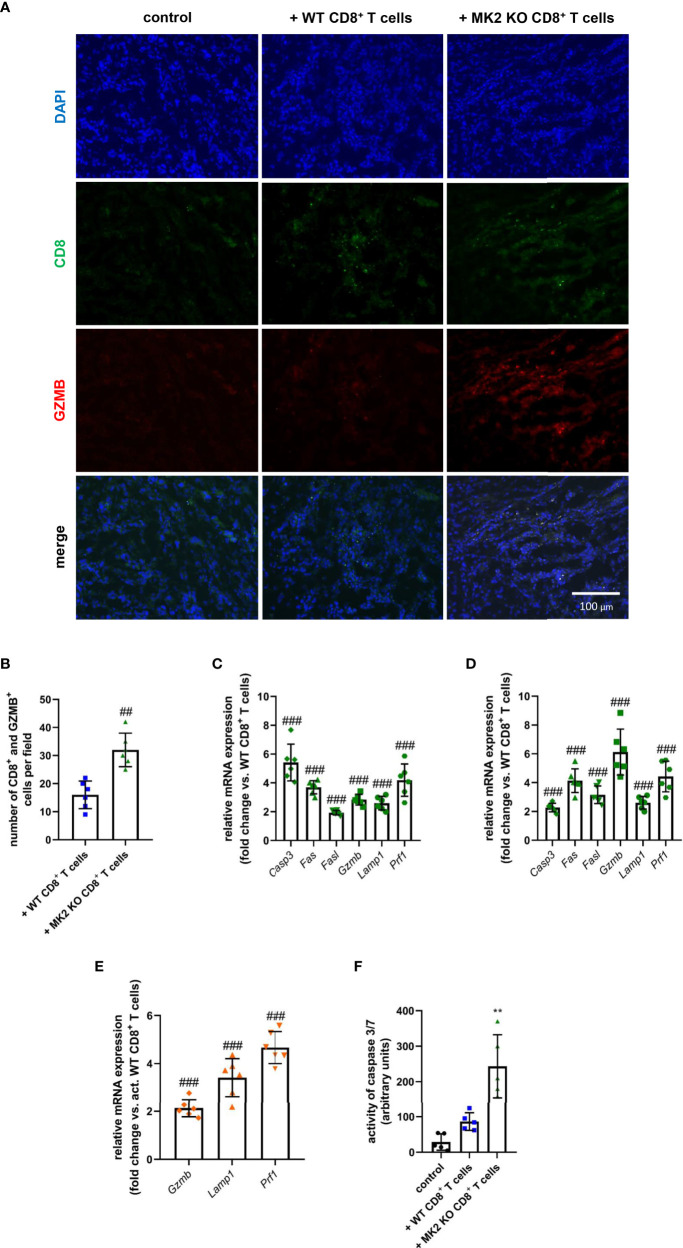
Tumors exposed to MK2 KO CD8^+^ T cells have increased cytotoxic and apoptotic factors. Representative images of immunofluorescence staining of CD8 and GZMB **(A)** and mean double positive cells **(B)** in PK5L1940 cell-derived allograft tumors obtained from untreated RAG1 KO mice and RAG1 KO mice treated with WT CD8^+^ T cells or MK2 KO CD8^+^ T cells. The expression of *Casp3*, *Fas*, *Fasl*, *Gzmb*, *Lamp1* and *Prf1* at the mRNA level in PK5L1940 **(C)** and BRAF **(D)** cell-derived allograft tumors obtained from RAG1 KO mice treated with MK2 KO CD8^+^ T cells is increased. The expression of *Gzmb*, *Lamp1*, *Prf1* at the mRNA level in activated MK2 KO CD8^+^ T cells **(E)** is enhanced. Caspase 3/7 activity **(F)** is increased in tumors as shown by fluorescence in BRAF tumor homogenates. Data are presented as means ± SD; 6 independent samples; **P < 0.01 vs. control, ^##^ *P* < 0.01, ^###^ *P* < 0.001 vs. + WT CD8^+^ T cells or activated WT CD8^+^ T cells.

Although MK2 is thought to be one of the regulators of immune response, especially in the context of macrophage action (13, 14, 17), its significance in the cytotoxic ability mediated by CD8^+^ T cells is still largely unexplored. In our study, increased CD8^+^ T cells were detected in tumors obtained from both animals treated with WT and MK2 KO CD8^+^ T cells compared to control mice, which was examined using immunofluorescence staining for CD8 (**Figure 2A**). Furthermore, increased granzyme B staining was detected in tumors with MK2 KO CD8^+^ T cells than in tumors with WT CD8^+^ T cells. The mean double positive cells per field indicated that there are more granzyme B expressing cells with MK2 KO CD8^+^ T cells (**Figure 2B**). Thus, we next investigated the expression of genes related to cytotoxicity and apoptosis in the tumors obtained from allograft models (**Figures 2C, D**). When apoptotic markers were examined in and PK5L1940 and BRAF tumors by real-time PCR, an increase of *Casp3* (from 2-fold to up to 6-fold) expression in animals treated with MK2 KO CD8^+^ T cells compared to animals treated with WT CD8^+^ T cells was observed (**Figures 2C, D**). Additionally, in tumors obtained from mice treated with MK2 KO CD8^+^ T cells, but not WT CD8^+^ T cells, genomic analysis documented increase expression of *Fas* and *Fasl*. It is worth noting that MK2 KO CD8^+^ T cell administration may not only affect apoptosis induced by FAS-mediated interaction, but, also are able to produce proteases killing gastrointestinal cancer cells. In fact, we noted that tumors obtained from mice treated with MK2 KO CD8^+^ T cells were characterized by significantly higher expression of *Gzmb* (from 3-fold to up to 6-fold) and *Prf1* (4-fold) when compared to animals treated with WT CD8^+^ T cells (**Figures 2C, D**). These findings are in line with the immunofluorescence described above. Additionally, our observation based on tumors analysis was confirmed by employing an *in vitro* approach, where we documented that *Gzmb*, *Lamp1*, and *Prf1* cytotoxic factors are increased in activated MK2 KO CD8^+^ T cells compared to activated WT CD8^+^ T cells from 2-fold to up to 4-fold (**Figure 2E**). As was shown in **Figure 2F**, in addition to CD8^+^ T cell activity and apoptotic genes, caspase 3/7 activity was increased in tumors treated with MK2 KO CD8^+^ T cells normalized by tumor weight suggesting an overall increase in apoptosis.

### 3.3 MK2 KO CD8^+^ T cells produce increased soluble anti-tumorigenic cytotoxic factors and cytokines

**Figure 3:**
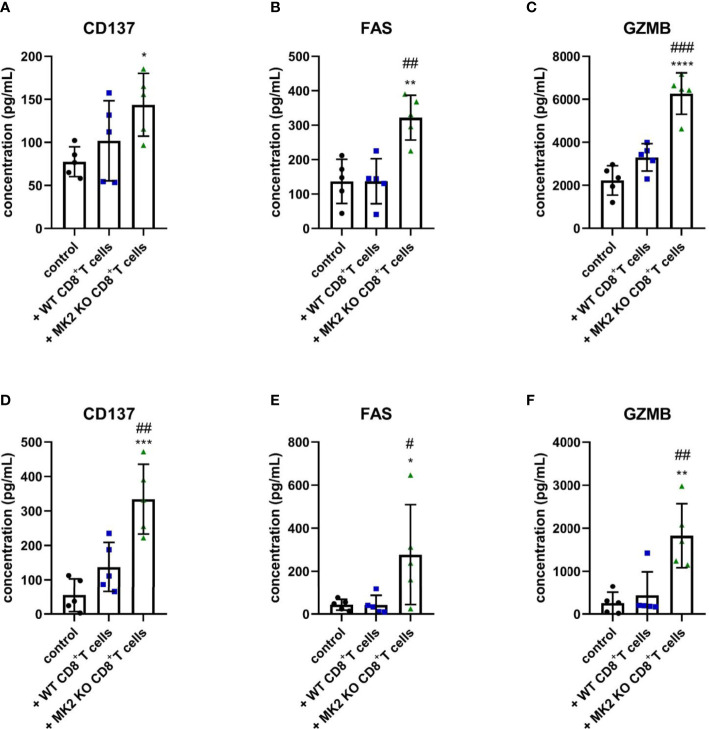
Tumors exposed to MK2 KO CD8^+^ T cells have increased anti-tumorigenic soluble factors. The concentration of CD137 **(A, D)**, FAS **(B, E)** and GZMB **(C, F)** in media from PK5L1940 **(A–C)** and BRAF **(D–F)** cell-derived allograft tumors obtained from untreated RAG1 KO mice and RAG1 KO mice treated with WT CD8^+^ T cells or MK2 KO CD8^+^ T cells is shown by multiplex array. Data are presented as means ± SD; 5 independent samples tested per group; ^*^ *P* <  0.05, ^**^ *P* <  0.01, ^***^ *P* <  0.001, ^****^ *P* < 0.0001 vs. control; ^#^ *P*<  0.05, ^##^ *P* <  0.01, ^###^ *P* < 0.001 vs. + WT CD8^+^ T cells.

The following results suggested that MK2 depletion in CD8^+^ T cells enhances expression of anti-tumorigenic factors in pancreas and colon cancers, we examined molecules released from PK5L1940 and BRAF cells-derived allograft tumors obtained from control mice and animals treated with WT or MK2 KO CD8^+^ T cells. As shown in **Figures 3A, D**, tumors obtained from mice treated with MK2 KO CD8^+^ T cells showed increased production of CD137, a marker of T cell activation. We further analyzed the tumor supernatants of both tumor types treated with MK2 KO CD8^+^ T cells compared to control and treated with WT CD8^+^ T cells and found soluble FAS and GZMB to be increased (**Figures 3B, C, E, F**). These data suggest that MK2 depletion in CD8^+^ T cells affects not only genomic changes in tumors but participates in secretome modulation, which inhibits the progression of gastrointestinal cancers.

**Figure 4:**
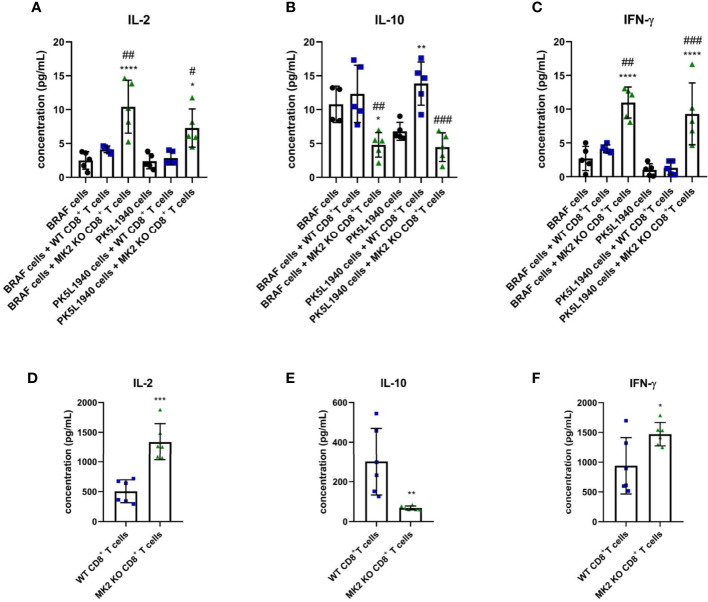
MK2 KO CD8^+^ T cells show changes in cytokine production. The concentration of IL-2 **(A)**, IL-10 **(B)** and IFN-γ **(C)** in media from PK5L1940 and BRAF cell-derived allograft tumors obtained from untreated RAG1 KO mice and RAG1 KO mice treated with WT CD8^+^ T cells or MK2 KO CD8^+^ T cells. The concentration of IL-2 **(D)**, IL-10 **(E)** and IFN-γ **(F)** in media obtained from activated WT and MK2 KO CD8^+^ T cells is shown by multiplex array. Data are presented as means ± SD; 5 – 6 independent samples tested per group; ^*^ *P* <  0.05, ^**^ *P* <  0.01, ^***^ *P* <  0.001, ^****^ *P* < 0.0001 vs. control; ^#^ *P*<  0.05, ^##^ *P* <  0.01, ^###^ *P* < 0.001 vs. + WT CD8^+^ T cells.

In addition to anti-tumorigenic/pro-apoptotic factors, changes in T cell cytokines were also observed between tumors treated with WT CD8^+^ T cells and tumors treated with MK2 KO CD8^+^ T cells. **Figures 4A–C** indicates that IL-2 and IFN-γ are highly produced in tumors treated with MK2 KO CD8^+^ T cells compared to tumors treated with WT CD8^+^ and controls, while IL-10 was significantly decreased. A similar phenomenon was observed *in vitro* where activated CD8^+^ T cells isolated from MK2 KO mice showed significantly increased secretion of IL-2 and IFN-γ and significantly decreased IL-10 production (**Figures 4D–F**). These data suggest that the MK2 pathway blockade enhances both cytotoxic factors and cytokines associated with T cell activity, while decreasing the inhibitory T cell cytokine, i.e. IL-10.

## 4 Discussion

Immunosuppressive cells such as tumor-associated macrophages, cancer-associated fibroblasts and regulatory T cells are one of the main sources of tumor-promoting signals in cancer. Accumulating evidence suggests that the immunosuppressive landscape of the tumor microenvironment has been linked with poor prognosis, worse clinical outcomes and decreased survival of patients with cancers (22–24). In progressive tumors, cytokines such as IL-10 or transforming growth factor β are highly produced and the function of antigen-presenting cells is disturbed. On the contrary, protective immunity against cancer mediated by CD8^+^ T cells is a main component of anti-tumor action of immune response. In fact, numerous reports have indicated that the amount and density of tumor infiltrating CD8^+^ T cells improve survival of patients with cancers (25–27). Nevertheless, limited efforts have been made to identify factors responsible for enhanced activity and improved anti-tumor function of CD8^+^ T cells.

The MK2 pathway has not been well examined in CD8^+^ T cells. Thus, here we provide the first evidence that MK2 is a significant serine/threonine kinase that regulates progression of gastrointestinal cancers through a mechanism related to cytotoxic function regulation of CD8^+^ T cells. Recently, observational and experimental studies have demonstrated that high expression of MK2 favors tumor progression (13–15, 17, 28). However, the link between MK2 and gastrointestinal cancers has not been investigated in the context of cytotoxic mechanisms induced by CD8^+^ T cells. There are studies suggesting that inhibition of p38 can reverse CD8^+^ T cell exhaustion by increasing proliferation pathways (29). However, since MK2 can act independently from p38, there is more to learn about its role in CD8^+^ T cell regulation.

In accordance with the above-mentioned evidence, we were able to identify molecular and cellular mechanisms of activity mediated by MK2 in cancer progression. The specific depletion of MK2 in CD8^+^ T cells led to a decrease in pancreas and colon tumor growth. Additionally, we found that gastrointestinal tumors obtained from mice treated with MK2 KO CD8^+^ T cells are characterized by increased expression of genes related to programmed cell death such as *Casp3*, *Fas* and *Fasl*. Among several caspases, CASP3 is a frequently activated protease leading to the specific cleavage of numerous proteins and coordinating the degradation of cellular structures (30). FAS-mediated apoptosis is another crucial mechanism of apoptosis, which is directly dependent on antigen-presenting cell interaction with cancer cells. In our study, we found that both apoptotic factors, i.e. FASL is overexpressed at the mRNA level and FAS at both the mRNA and protein levels in tumors obtained from mice treated with MK2 KO CD8^+^ T cells, suggesting the functional significance of MK2 in CD8^+^ T cells in combating gastrointestinal cancers.

Nevertheless, it should be noted that the increased anti-tumorigenic activity of CD8^+^ T cells with MK2 KO is not only associated to apoptosis induced by CASP3 and/or FAS-FASL machinery, but also affects proteases, which are secreted by CD8^+^ T cells. In our study we noted that stimulated MK2 KO, but not WT CD8^+^ T cells are characterized by enhanced gene expression of lytic molecules such as GZMB and PRF1. Cell death induced by the above-mentioned factors has been observed as a primary mechanism that is used by CD8^+^ T cells to eliminate cancer cells. On the other hand, not only membrane-bound granules, but also surface-related lytic granules contain lysosomal-associated glycoproteins (LAMPs) such as LAMP1 (known as a CD170a) are expressed in activated T cells. According to the evidence presented by Betts et al., cell surface accumulation of LAMP1 is indicative of CD8^+^ T cell activity in an antigen-specific manner (31). Although we did not address antigen specificity here, the ability of CD8^+^ T cells to decrease tumor size *in vivo* without prior activation suggests that splenic CD8^+^ T cells are able to recognize tumor cells.

Finally, secretome analysis confirmed our genomic findings and revealed that MK2 acively particpates in the regulation of cytotoxic function of T cells. We found that CD8^+^ T cells with MK2 KO are characterized by ehnanced activity, which was shown by multiplex analysis of CD137 concentrations in pancreas and colon tumor supernatants. CD137 is a sensitive marker of acivated CD8^+^ T cells and is not expressed on resting cells, suggesting that CD137 can be used in the clinical practice to monitor immunotherapy efficency (32). In our study, we found an increased concentration of CD137 in tumors obtained from mice with gastroinestinal cancers and treated with MK2 KO CD8^+^ T cells when compared to animals treated with WT CD8^+^ T cells and control group. This *in vivo* evidence suggests that MK2 affects activity of CD8^+^ T cells and MK2 can be targeted in the immunotherapy of gastrointestinal cancers.

The MK2 pathway is most well known for regulation of cytokine production by various cell types (13, 14, 17, 18). However, the role of the MK2 pathway in regulation of cytokine expression in T cells has not been examined. Here we found that MK2 knockdown in CD8^+^ T cells led to increased IL-2 and IFN-γ production in the tumor microenvironment and *in vitro.* IL-2 plays a crucial role in the polarization, expansion and survival of T cells (33, 34). Moreover, CD8^+^ T cells stimulated by IL-2 are manifested by enhanced cytotoxicity and are able to induce apoptosis employing FAS-FASL machinery (35). On the other hand, IFN-γ is another major component of anti-tumorigenic immunity with a half-life that is much longer than the half-life of IL-2 in the blood (36, 37). The action of IFN-γ is not only directly related to the regulation of apoptosis but IFN-γ can indireclty modulate the function of endothelial cells, as well as immunce cells in the tumor microenviroment. For instance, the cancer regression mediated by IFN-γ affects angiogenesis inhibition and macrophage polarization (38, 39). Martini et al. documented that IFN-γ increases the expression of MHC class I molecules in cancer cells improving cancer cell exposition for antigen-presenting cells (40). Here we provide evidence that activated CD8^+^ T cells with MK2 depletion are manifested by anti-tumorigenic phenotype of CD8^+^ T cells. Moreover, our study is the first to indicate a link between MK2 and IL-2 production in T cells were noted. Slightly higher production of IL-2 and IFN-γ in mice treated with WT CD8^+^ T cells compared to control mice suggested impact of the tumor microenvironment on CD8^+^ T cells. On the other hand, in activated MK2 KO CD8^+^ T cells with there was a concurrent decrease in IL-10 production similar to that we and others have seen by macrophages (14, 17). IL-10 is a tumor-promoting cytokines and its down-regulation suggests that CD8^+^ T cells without MK2 are potent dirver of anti-tumorigenic action and promotes gastrointestinal tumors regression.

Collectivelly, comaprision of immune respons generated by WT CD8^+^ T cells and CD8^+^ T cells with MK2 depletion showed a major impact of MK2 expression and sigalling on anti-tumor activity and function of CD8^+^ T cells. Additionally, we were able to prove that enhanced immune signals related with MK2 and mediated by CD8^+^ T cells lead to increased apoptotic markers and inhibition of gastroinetstinal cancer growth. In our study, RAG1 KO mice were utilized to exmaine the specific impact of MK2 in CD8^+^ T cells on GI tumors. Further studies with animals with complete components of immune response should be employed to explore the impact of other cell types in this system. Furthermore, we did not pre-active the cells, thus allowing the cells to be activated by the tumor microenvironemnt. However, to further enhance the activity of MK2 KO CD8^+^ T cells, pre-activation could be employed. Taken together, our evidence highlighted that immunothepapy based on MK2 expresssion and signalling modulation can be effective and indirect approach targeting tumor micoenviroment. Thus, there is potential for therapy utilizing MK2 inhibition that could be combined with other immunotherapies in order to combat tumor progression.

## Data availability statement

The raw data supporting the conclusions of this article will be made available by the authors, without undue reservation.

## Ethics statement

The animal study was reviewed and approved by The research has been approved by the University of Utah Institutional Animal Care and Use Committee.

## Author contributions

All authors listed have made a substantial, direct, and intellectual contribution to the work and approved it for publication.
